# Novel Human Bispecific Aptamer–Antibody Conjugates for Efficient Cancer Cell Killing

**DOI:** 10.3390/cancers11091268

**Published:** 2019-08-29

**Authors:** Margherita Passariello, Simona Camorani, Cinzia Vetrei, Laura Cerchia, Claudia De Lorenzo

**Affiliations:** 1 Department of Molecular Medicine and Medical Biotechnology, University of Naples “Federico II”, Via Pansini 5, 80131 Napoli, Italy; 2 Ceinge—Biotecnologie Avanzate s.c. a.r.l., via Gaetano Salvatore 486, 80145 Naples, Italy; 3 Institute of Experimental Endocrinology and Oncology “Gaetano Salvatore” (IEOS), CNR, Via S. Pansini 5, 80131 Napoli, Italy

**Keywords:** antibody, aptamer, cancer, immunotherapy, dual targeting

## Abstract

Monoclonal antibodies have been approved by the Food and Drug Administration for the treatment of various human cancers. More recently, oligonucleotide aptamers have risen increasing attention for cancer therapy thanks to their low size (efficient tumor penetration) and lack of immunogenicity, even though the short half-life and lack of effector functions still hinder their clinical applications. Here, we demonstrate, for the first time, that two novel bispecific conjugates, consisting of an anti-epidermal growth factor receptor (EGFR) aptamer linked either with an anti-epidermal growth factor receptor 2 (ErbB2) compact antibody or with an immunomodulatory (anti-PD-L1) antibody, were easily and rapidly obtained. These novel aptamer–antibody conjugates retain the targeting ability of both the parental moieties and acquire a more potent cancer cell killing activity by combining their inhibitory properties. Furthermore, the conjugation of the anti-EGFR aptamer with the immunomodulatory antibody allowed for the efficient redirection and activation of T cells against cancer cells, thus dramatically enhancing the cytotoxicity of the two conjugated partners. We think that these bispecific antibody–aptamer conjugates could have optimal biological features for therapeutic applications, such as increased specificity for tumor cells expressing both targets and improved pharmacokinetic and pharmacodynamic properties due to the combined advantages of the aptamer and antibody.

## 1. Introduction

Immunotherapy based on the use of novel human monoclonal antibodies (mAbs) with antitumor or immunomodulatory activity is an increasingly important strategy for cancer management [1]. MAbs can be directed against tumor-associated antigens (TAA), overexpressed on the cell surface of tumor cells, to either inhibit their oncogenic function or to specifically deliver toxic compounds. Alternatively, mAbs can be used in cancer therapy to regulate specific T-cell responses, thus enhancing the protective role of the immune system against cancer [2].

We have produced both human anti-TAA and immunomodulatory antibodies useful for breast cancer therapy. A validated target for breast cancer immunotherapy is ErbB2, a tyrosine kinase receptor (TKR) overexpressed on many carcinoma cells with a key role in the development of malignancies [3,4,5].

Trastuzumab, a humanized anti-ErbB2 antibody, is effective in the therapy of breast carcinoma, but it engenders cardiotoxicity and many cancer patients are resistant to Trastuzumab treatment [6,7,8]. Antitumor strategies based upon biologics have been further successfully established for ErbB2 TKR, leading to the recent clinical approval of Pertuzumab and TDM-1 [9,10]. Unfortunately, the clinical efficacy of these drugs is still limited by resistance and cardiotoxicity issues [11,12].

Novel human antitumor conjugates were engineered in our laboratory by isolation, through phage display technology of novel human antibody fragments, such as Erbicin [13], a human anti-ErbB2 scFv, and its fusion with either a human RNase [14], the Fc region of a human IgG1 (Erb-hcAb) [15], or with other scFvs targeting different epitopes of ErbB2 [16] or targeting CD3 for recruiting T cells [17]. Erbicin-derived immunoagents (EDIAs) were found to be selectively cytotoxic for ErbB2-positive cancer cells (including those resistant to Trastuzumab) in vitro and in vivo, to recognize an epitope different from that of Trastuzumab and Pertuzumab [18], and not to be cardiotoxic [19,20].

On the other hand, cancer immunotherapy based on immunomodulatory antibodies is becoming a mainstay of modern oncology. Checkpoint inhibitors (CIs), i.e., anti-Programmed Death Receptor/Ligand-1 (PD-1/PD-L1) and anti-cytotoxic T-lymphocyte antigen 4 (CTLA-4) agents, have achieved unprecedented efficacy with long-lasting clinical benefit. These antibodies are currently used for the treatment of several tumors [21,22,23,24]; however, treatment efficacy with CIs in monotherapy is still limited to 20–30% of the population [25,26,27].

Among the immune checkpoints, the PD-1/PD-L1 pathway could represent an attractive target as it has been reported not only to inhibit human T cell responses, thus promoting immune evasion of tumor cells [28], but also to induce tumor cell proliferation [29]. Immunomodulatory antibodies-based treatment, targeting the PD-1/PDL-1 pathway, displayed antitumor responses in patients with several cancer types, such as advanced melanoma, non-small cell lung cancer, or breast cancer [30]. Cancer cells can develop an immunosuppressive microenvironment for evasion from immune surveillance upon tyrosine kinase inhibitor (TKI) intervention, even though the detailed mechanisms of acquired immune evasion are not yet understood [31]. Indeed, the increased expression of PD-L1 was also observed in epidermal growth factor receptor (EGFR)-resistant tumors, suggesting that the activation of the immune checkpoint may delay the treatment efficacy of TKIs in cancer patients [31].

Hence, EGFR and PD-L1 could be considered as potential co-targets for combinatorial breast cancer treatments [32,33]. Furthermore, recent studies have demonstrated that the activation of EGFR modulates not only the intracellular pathways involved in tumor cell survival of many cancer types, but also the host antitumor immunity, by driving PD-L1 expression. Indeed, EGFR signaling can induce direct or indirect PD-L1 upregulation, inducing immune escape, whereas the inhibition of EGFR signaling downregulates PD-L1 expression [34,35,36,37,38,39].

As previously reported, combinatorial antibody treatments against different targets induce synergistic effects [40]; in particular, several clinical studies combining PD-L1/PD-L1 inhibitors with either ErbB2 or EGFR-TKIs in cancer patients are on-going [35,39,41]. To this aim, we decided to test whether novel combinations of immunomodulatory antibodies with other highly selective compounds emerging for anti-cancer therapy, such as oligonucleotide aptamers [42,43] targeting TKRs, could increase the antitumor efficacy. Aptamers are single-stranded oligonucleotides that, similarly to antibodies, interact at high affinity and specificity with their targets. They offer unique chemical and biological characteristics: small size, high stability, convenient synthesis, and modification with minimal inter-batch variability and lack of immunogenicity. They have been used as cancer therapeutics because of their ability to inhibit their targets and, more recently, as carriers for cell-targeted delivery of therapeutic secondary reagents. A nuclease-resistant RNA-aptamer able to recognize EGFR has been generated in our laboratory, which inhibits proliferation in vitro and in animal models of non-small cell lung cancer [44], glioblastoma [45,46], and triple-negative breast cancer (TNBC) [47]. Furthermore, the aptamer has been validated as targeting ligand to deliver therapeutic anti-microRNA (miRNA) to EGFR-positive TNBC implanted in mice [48]. Herein, we have investigated the efficacy of combinatorial treatments of the anti-EGFR aptamer with anti-ErbB2 and anti-PD-L1 antibodies. Highly relevant to the goals of this plan was the assumption that the different molecular structure and mechanism of action of aptamers and antitumor immunoagents could be exploited for the first time to achieve unprecedented biological effects, as compared to individual agents.

Given the possibility of introducing structural changes that make aptamers available to the conjugation with secondary reagents, we developed innovative and efficacious chimeric molecules consisting of the anti-EGFR aptamer and anti-ErbB2 or anti-PD-L1 antibody that could allow to overcome the limits of aptamers, such as short half-life in circulation, lack of effector functions, and reduced biostability.

## 2. Results

### 2.1. Evaluation of the Effects on Tumor Cells of Combined Treatments of Erb-hcAb with Anti-EGFR Aptamer

It has been reported that combinatorial treatment of human cancers with anti-ErbB2 and anti-EGFR antibodies leads to more effective growth inhibition than either treatment alone [49,50]. On the basis of this observation, we have investigated the possibility to use the anti-ErbB2 compact antibody (Erb-hcAb) [15] in combination with the anti-EGFR aptamer (CL4) [44] to ascertain whether the combined treatments allow for more effective and lower therapeutic doses of these drugs.

**Figure 1:**
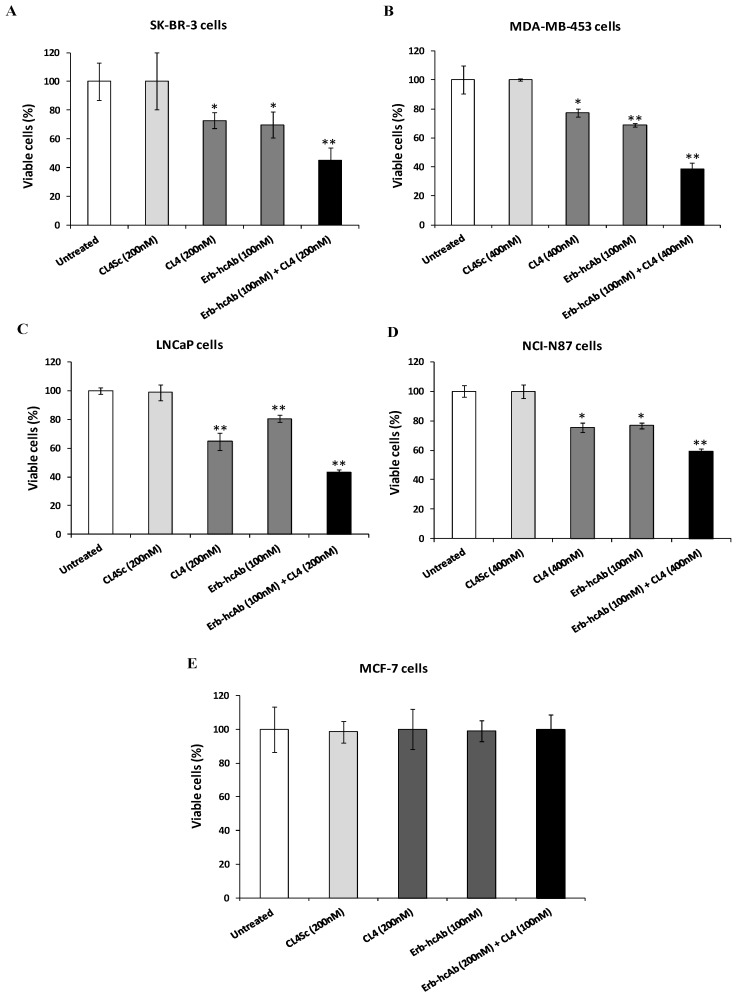
Combined treatment of Erb-hcAb and CL4 selectively inhibits tumor cell survival. SK-BR-3 (**A**), MDA-MB-453 (**B**), LNCaP (**C**), NCI-N87 (**D**), and MCF-7 (**E**) cells were treated for 72 h with Erb-hcAb or CL4, alone or in combination, at the indicated concentrations. Cell survival after treatments is expressed as percentage of viable treated cells with respect to untreated cells. The scrambled aptamer (CL4Sc) was used in parallel as a negative control. Error bars depict means ± SD. *p*-values for the indicated mAbs relative to untreated cells, and for CL4 relative to CL4Sc, are: ** *p* < 0.01; * *p* < 0.05.

**Figure 2:**
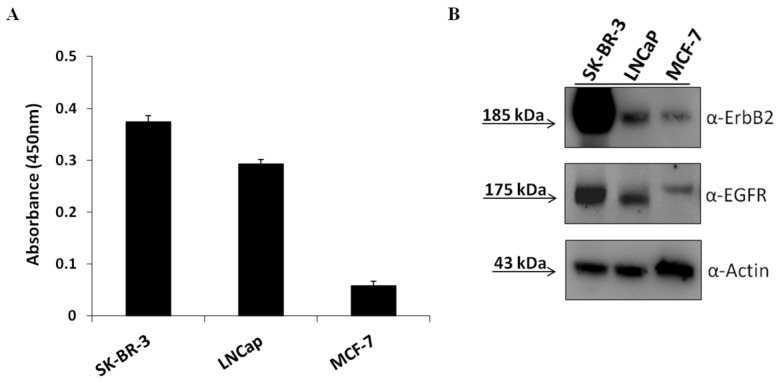
Expression of ErbB2, EGFR, and PD-L1 on tumor cell lines. Cell ELISA assay with a commercial anti-PD-L1 antibody on SK-BR-3, LNCaP, and MCF-7 tumor cells (**A**) for detection of cell surface PD-L1 expression. Western blotting analyses with the commercial anti-ErbB2 and anti-EGFR mAbs of extracts from SK-BR-3, LNCaP, and MCF-7cells. The intensity of the bands was normalized to actin (**B**). The ratios of ErbB2/actin and EGFR/actin signal intensities were calculated for each cell extract and found to be about 30 and 5 for SK-BR-3, 2 and 3 for LNCaP and 0.2 and 0.3 for MCF-7, respectively.

To this aim, EGFR- and ErbB2-positive cancer cells, including breast (SK-BR-3 and MDA-MB-453), prostate (LNCaP), and gastric (NCI-N87) cancer cell lines [51,52,53,54], were treated with a combination of Erb-hcAb and CL4 at increasing concentrations to test whether the inhibitors affect cell viability more efficiently than when they are used as single agents. As shown in Figure 1 and Supplementary Figure S1, the combined treatment led to a stronger reduction of cell viability compared to single receptor blocking (60% growth inhibition on SK-BR-3, MDA-MB-453, and LNCaP, and 40% on NCI-N87 cells after combinatorial treatments with respect to about 20–30% after their use in monotherapy). As a negative control, we used in parallel assays mammary MCF-7 cells (Figure 1E) expressing very low levels of EGFR and ErbB2, as shown in the Western blotting analysis of cell extracts (see Figure 2), and no significant effects were observed either in single or combinatorial treatments, thus confirming the binding specificity of the compounds for their targets.

### 2.2. Evaluation of the Effects on Tumor Cell Viability of Combined Treatments of Anti-PD-L1 mAb with Anti-EGFR Aptamer

Several clinical studies combining PD-1/PD-L1 pathway inhibitors with EGFR inhibitors in cancer patients are on-going [41].

**Figure 3:**
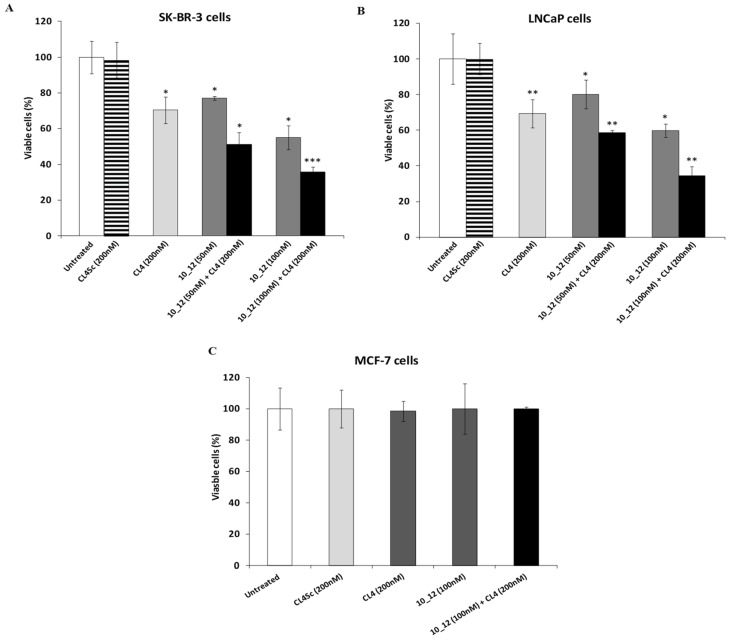
Combined treatment of CL4 and anti-PD-L1 mAb efficiently inhibits tumor cell survival. SK-BR-3 (**A**), LNCaP (**B**), and MCF-7 (**C**) cells were treated for 72 h with CL4 or 10_12 mAb, alone or in combination, at the indicated concentrations. Cell survival is expressed as percent of viable treated cells with respect to untreated cells. CL4Sc was used in parallel as a negative control. Error bars depict means ± SD. *p*-values for the indicated mAbs relative to untreated cells, and for CL4 relative to CL4Sc, are: *** *p* ≤ 0.001; ** *p* < 0.01; * *p* < 0.05.

PD-L1 expression has been found to be upregulated by EGFR overexpression in several types of cancer cells, suggesting us to investigate on a dual EGFR and PD-L1 targeting strategy. To this aim, we first tested the effects on cancer cell viability of the anti-EGFR CL4 aptamer in combination with a human anti-PD-L1 mAb named 10_12 [55] to then verify whether a bispecific construct made up of these two moieties could be considered beneficial for anti-cancer treatment. We chose SK-BR-3 and LNCaP cancer cells as models since they express both EGFR and PD-L1 (see Figure 2) on their surface [52,54,56,57]. The MCF-7 mammary cell line, expressing low levels of cell surface EGFR and PD-L1, was used as a negative control. As shown in Figure 3, the anti-PD-L1 antibody significantly inhibited the growth of both the PD-L1-positive cell lines tested and, importantly, the combined treatment with CL4 led to additive effects, whereas no significant effects were observed on MCF-7 cells for both single and combined treatments (Figure 3 and Supplementary Figure S2). The immune independent antitumor activity of anti-PD-L1 mAb was previously ascribed to its ability to affect the mitogen-activated protein kinases (MAPKs) pathway in tumor cells [58].

**Figure 4:**
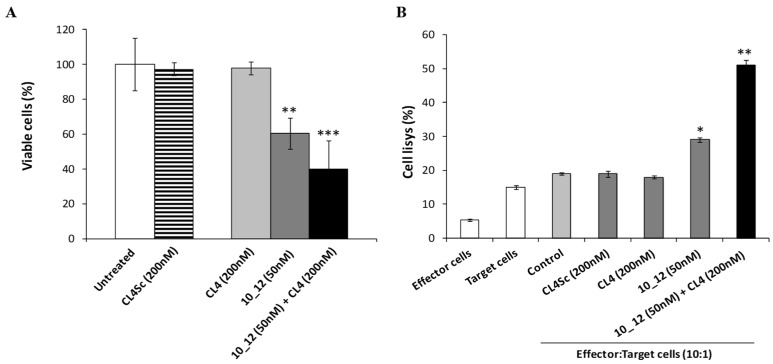
Cytotoxic effects of the combination of CL4 aptamer and 10_12 mAb on breast cancer cells co-cultured with lymphocytes. (**A**) SK-BR-3 cells were co-cultured with lymphocytes (effector:target ratio 10:1) left untreated (control) or treated for 24 h with CL4 or 10_12, used alone or in combination, at the indicated concentrations. SK-BR-3 cell survival is expressed as percentage of viable treated cells with respect to untreated cells. (**B**) SK-BR-3 cell lysis, measured by the LDH release after the incubation with CL4 or 10_12 compounds, used at the indicated concentrations. The levels of LDH are expressed as a percentage of cell lysis with respect to the effects observed in co-cultures of tumor cells and lymphocytes in the absence of the drugs, used as a control. In (**A**,**B**), error bars depict means ± SD. *p-*values for the indicated mAbs relative to untreated cells, and for CL4 relative to CL4Sc, are: *** *p* ≤ 0.001; ** *p* < 0.01; * *p* < 0.05.

Furthermore, the efficacy of this combinatorial approach was also tested on SK-BR-3 breast tumor cells when co-cultured with human lymphocytes to exploit also the inhibitory effects of 10_12 mAb in the PD-1/PD-L1 interaction [15,59]. Indeed, the 10_12 mAb is an affinity-matured variant (containing three single point mutations in the heavy chain CDR3) of the anti-PD-L1 mAb, called PD-L1_1, which was previously found to specifically activate CD3-positive T cells by FACS analyses of treated human peripheral blood mononuclear cells (hPBMCs) [60]. To this aim, SK-BR-3 cells were treated with CL4 aptamer (200 nM) or 10_12 mAb (50 nM), used alone or in combination, in the absence or in the presence of hPBMCs (effector: target ratio 10:1) for 24 h at 37 °C. As shown in Figure 4A and Supplementary Figure S3, the presence of lymphocytes induced a drastic reduction of cancer cell viability, leading to 60% of cell death when CL4 and 10_12 were used in combination. Cells left untreated or treated with the scrambled CL4Sc aptamer were used as controls.

The cytotoxic effect on target cells was also analyzed in parallel by lactate dehydrogenase (LDH) assays, performed on cell culture media. As a sign of cell lysis, we measured the levels of LDH [61] released from cells co-cultured with lymphocytes and treated for 24 h with CL4 aptamer or 10_12 mAb, used alone or in combination. The results shown in Figure 4B confirm that the combinatorial treatment in the presence of lymphocytes induces higher levels of LDH release corresponding to 60% of cells lysis, thus showing a more potent effect than that observed with either the single anti-PD-L1 antibody or anti-EGFR aptamer.

Since these treatments could improve T cell effector functions against cancer cells due to the inhibitory effects of 10_12 mAb in the PD-1/PD-L1 interaction [59] between tumor cells and T cells, we also verified the ability of the indicated compounds to induce the secretion of cytokines by lymphocytes involved in immunological surveillance.

**Figure 5:**
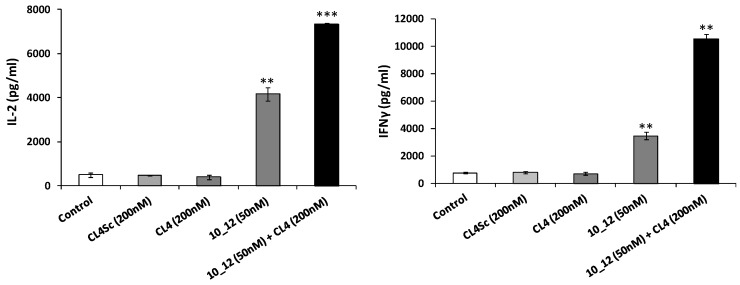
Effects of CL4 aptamer and 10_12 mAb on secretion of cytokines from lymphocytes co-cultured with breast cancer cells. SK-BR-3 cells were co-cultured with lymphocytes (effector:target ratio 10:1) and treated for 24 h at 37 °C with CL4 aptamer or 10_12 mAb, used alone (grey column) or in combination (black column), at the indicated concentrations. IL-2 and IFNγ levels (pg/mL) were obtained by ELISA assays performed on cell supernatants. Cells untreated (control) or treated with CL4Sc in the presence of lymphocytes were used as negative controls. Error bars depict means ± SD. *p-*values for the indicated mAbs relative to untreated cells are: *** *p* ≤ 0.001; ** *p* < 0.01.

As reported in Figure 5, a significant increase in the secretion of IL-2 and IFNγ cytokines was observed when PD-L1/EGFR positive SK-BR-3 cells were incubated for 24 h with lymphocytes and treated with the combination of CL4 and 10_12 with respect to that observed with the single agent treatments.

Altogether, these findings suggest that bispecific constructs made up of the anti-PD-L1 mAb and CL4, or Erb-hcAb and CL4, could become more potent antitumor agents than the parental mAbs [31].

### 2.3. Construction and Purification of the New Bispecific Constructs Made up of Erb-hcAb or 10_12 mAb Conjugated with the Anti-EGFR Aptamer

**Figure 6:**
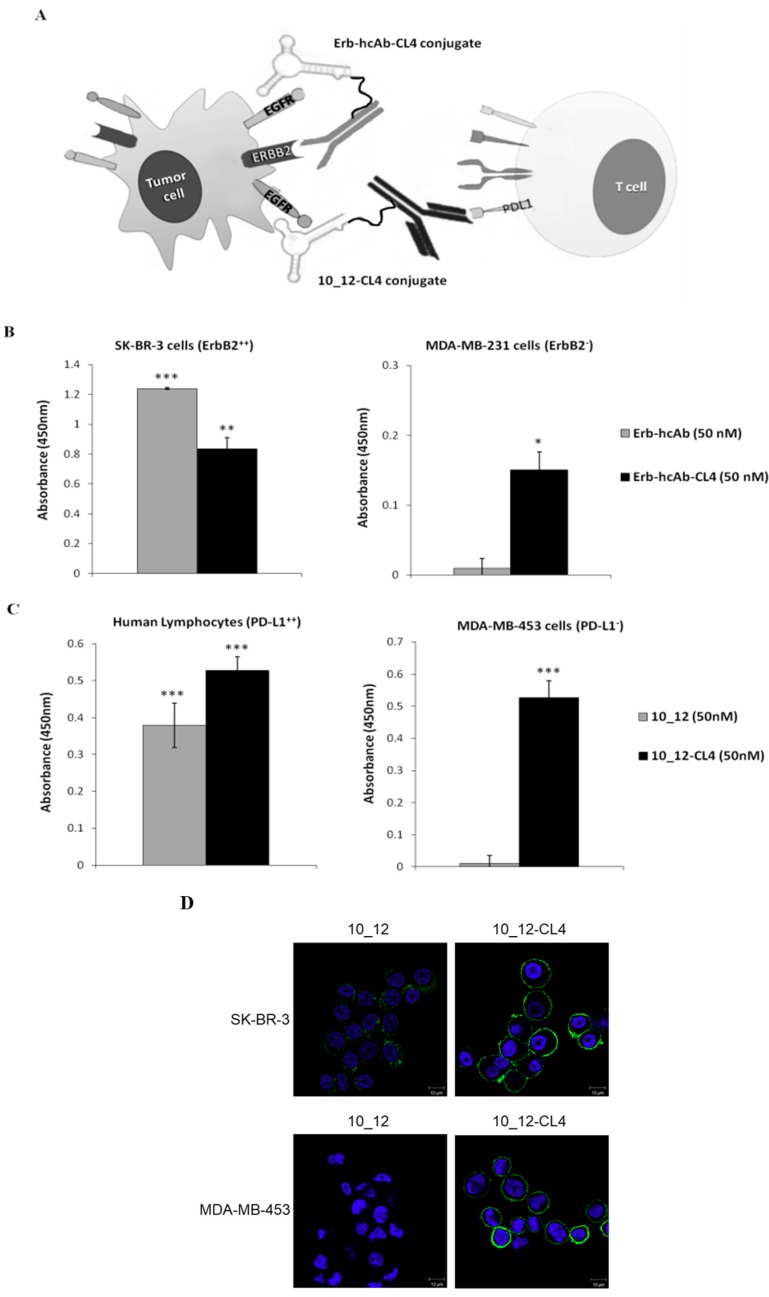
Schematic representations and binding of Erb-hcAb-CL4 and 10_12-CL4 conjugates. (**A**) Schematic representations of Erb-hcAb-CL4 (grey) or 10_12-CL4 (black) conjugates, generated by the fusion of CL4 amino-oligonucleotide (anti-EGFR aptamer) with the Fc region of Erb-hcAb (anti-ErbB2 compact antibody) or 10_12 (anti-PD-L1 monoclonal antibody). (**B**) Binding of Erb-hcAb-CL4 conjugate to SK-BR-3 or MDA-MB-231 cells. As controls, unconjugated Erb-hcAb antibody and CL4 aptamer were tested in parallel by ELISA assays at the same concentration. (**C**) Binding of 10_12-CL4 to MDA-MB-453 cells and human activated lymphocytes. As a control, unconjugated 10_12 antibody was tested in parallel by ELISA assays at the same concentration. Error bars depict means ± SD. In B and C, *p*-values for the indicated mAbs relative to untreated cells, are: *** *p* ≤ 0.001; ** *p* < 0.01; * *p* < 0.05. (**D**) Representative confocal microscopy images of SK-BR-3 and MDA-MB-453 cells incubated with 10_12, 10_12-CL4, as indicated. 10_12 and10_12-CL4 are visualized in green. Nuclei are visualized in blue. Magnification 63×, 1.0× digital zoom. Scale bar = 10 µm.

To generate the antibody–aptamer constructs, we conjugated the constant region of the antibody with the aptamer through a free amino group attached at the 5′ end of the 2′FPy-containing RNA sequence (Figure 6A), by exploiting the well-known coupling chemistry of formylbenzamide (4FB). In order to minimize the steric hindrance between the two moieties of the chimeric construct, we inserted a six-carbon spacer arm in the aptamer.

Specifically, the amino-terminated 2′FPy RNA aptamer was conjugated with the bifunctional succinimidyl-4-formylbenzamide linker (*S*-*4FB*), which incorporated an aromatic aldehyde functional group (formylbenzamide, 4FB) at the 5′end of the oligonucleotide, and then reacted with the S-HyNic-modified Erb-hcAb immunoagent or 10_12 mAb. The conjugation allowed to proceed for 2 h by gentle rotation at room temperature (RT) following the manufacturer’s recommendations [62].

Briefly, the antibodies were modified by using the S-HyNic reagent and mixed with the 4FB-modified aptamer in the presence of a reaction catalyst (aniline) to form the conjugate through a covalent bond. The conjugates, named Erb-hcAb-CL4 and 10_12-CL4, were then purified by a magnetic affinity matrix and eluted following the manufacturer’s recommendations (see Materials and Methods).

### 2.4. Characterization of Antibody–Aptamer Conjugates

First, we assessed whether, in the frame of the chimeric molecules, the parental antibodies preserve the ability to recognize their proper ErbB2 or PD-L1 receptor target. To this aim, Erb-hcAb-CL4 and 10_12-CL4 conjugates (50 nM final concentration) were incubated for 75 min at RT onto SK-BR-3 cells (ErbB2-positive cells) [54] and human lymphocytes (PD-L1-positive) [35], respectively. Binding was measured by enzyme-linked immunosorbent assay (ELISA) assays, and compared to that of unconjugated antibodies. As shown, a statistically significant binding was observed with the conjugates on the respective cell line tested (Figure 6B,C), thus indicating that the covalent attachment of the aptamer to the antibody, in the frame of both the conjugates, did not affect the antibody targeting function.

Notably, the Erb-hcAb-CL4 and 10_12-CL4 conjugates, differently from their antibody counterparts, were also able to bind to MDA-MB-231 and MDA-MB-453 cells, lacking ErbB2 and PD-L1 expression [63], respectively, thus indicating that the conjugates’ binding is mediated by the aptamer moiety, which retains the ability to bind to EGFR expressed on both the cell lines (Figure 6B,C). As a control, we also tested the combination of unconjugated CL4 aptamer and 10_12 mAb on MDA-MB-453 cells and, as expected, no significant binding to the cell line tested was detected with the anti-human IgG H+L, thus further confirming that the binding of 10_12 mAb to the EGFR-positive cell line occurs only when the molecule is covalently conjugated to the aptamer, as expected. Accordingly, confocal microscopy analyses showed that the 10_12-CL4 conjugate efficiently localized on the membrane of both SK-BR-3 and MDA-MB-453 cells, whereas the parental antibody recognized only PD-L1-positive SK-BR-3 cells [56] (Figure 6D).

These results demonstrate that the antibody and aptamer moieties maintain their targeting functions in the chimeric proteins.

### 2.5. Cytotoxic Effects of the Novel Aptamer–Antibody Conjugates on Tumor Cells

The finding that the novel immunoconjugates are endowed with both binding specificities for their targets led us to investigate whether they could also inhibit the growth of cells overexpressing both the targets more efficiently than the parental antibody or aptamer, confirming the results of combinatorial treatments reported above.

To this aim, SK-BR-3 and LNCaP cells (expressing ErbB2, EGFR and PD-L1) [52,54,56,57] were plated in the absence or in the presence of the immunoconjugates, used at increasing concentrations, and incubated for 72 h. Cell survival was measured by counting trypan blue-excluding cells. In parallel experiments, the effects on cell survival of the parental antibodies and anti-EGFR aptamer were also tested. We used lower concentrations (down to 50 nM) of the conjugate with respect to those (200–400 nM) used for the aptamer in combinatorial treatments, reported above, to verify whether the doses could be lowered for eventual therapeutic applications.

**Figure 7:**
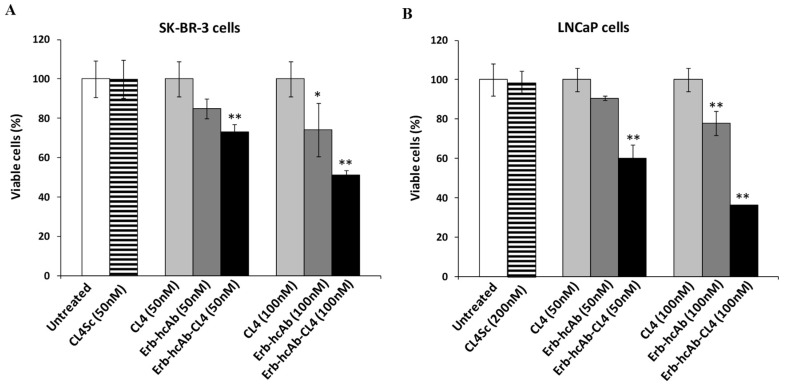
Effects of Erb-hcAb-CL4 conjugate on tumor cell survival. SK-BR-3 (**A**) and LNCaP (**B**) cells were incubated for 72 h in the absence or in the presence of CL4, Erb-hcAb, or Erb-hcAb-CL4 conjugate at the indicated concentrations. Cell survival is expressed as percentage of viable treated cells with respect to untreated cells. The scrambled aptamer was used in parallel as a negative control. Error bars depict means ± SD. *p-*values for the indicated mAbs relative to untreated cells are: ** *p* < 0.01; * *p* < 0.05.

**Figure 8:**
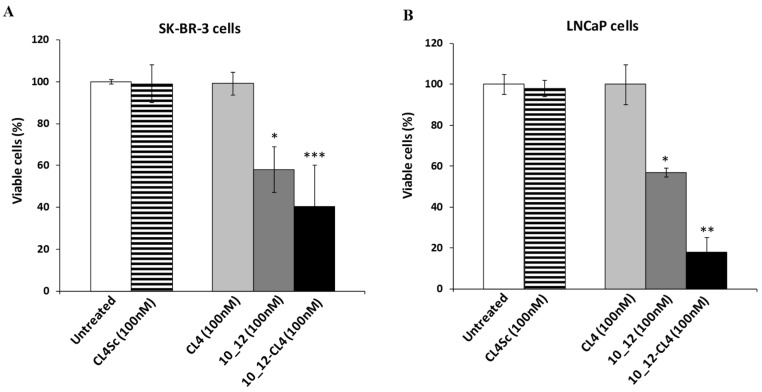
Effects of 10_12-CL4 conjugate on tumor cell survival. SK-BR-3 (**A**) and LNCaP (**B**) cells were incubated for 72 h in the absence or in the presence of CL4, 10_12, or 10_12-CL4 conjugate at the indicated concentrations. Cell survival is expressed as percentage of viable treated cells with respect to untreated cells. The scrambled aptamer was used in parallel as a negative control. Error bars depict means ± SD. *p-*values for the indicated mAbs relative to untreated cells are: *** *p* ≤ 0.001; ** *p* < 0.01; * *p* < 0.05.

As shown, Erb-hcAb-CL4 (Figure 7 and Supplementary Figure S4) and 10_12-CL4 (Figure 8 and Supplementary Figure S5) conjugates were found to be selectively cytotoxic for both tumor cell lines in a dose-dependent manner. The IC50 values were found to be lower than those obtained for CL4 and parental antibodies used as single agents, thus confirming that the novel immunoconjugates have more potent cytotoxic effects than the single antibodies and aptamer. These results strongly indicate that bispecific constructs made up of antitumor antibody and aptamer could become valuable tools for increasing the antitumor efficacy.

Finally, the efficacy of 10_12-CL4 was also tested on SK-BR-3 cells co-cultured with human lymphocytes to exploit also the inhibitory effects of 10_12 mAb in the PD-1/PD-L1 interaction [15,59] between tumor cells and T cells, which activates T cells by inducing IL2 and IFNγ cytokines secretion, as reported above and further detailed in another manuscript [55].

To this aim, SK-BR-3 cells were treated with the immunoconjugate (50 nM) or the unconjugated CL4 aptamer and 10_12 mAb (50 nM), used alone or in the presence of hPBMCs (effector:target ratio 10:1) for 24 h at 37 °C. We used lower concentrations (down to 50 nM) of the conjugate with respect to those (200–400 nM) used for the aptamer in combinatorial treatments, reported above, to verify whether the doses could be lowered for eventual therapeutic applications.

**Figure 9:**
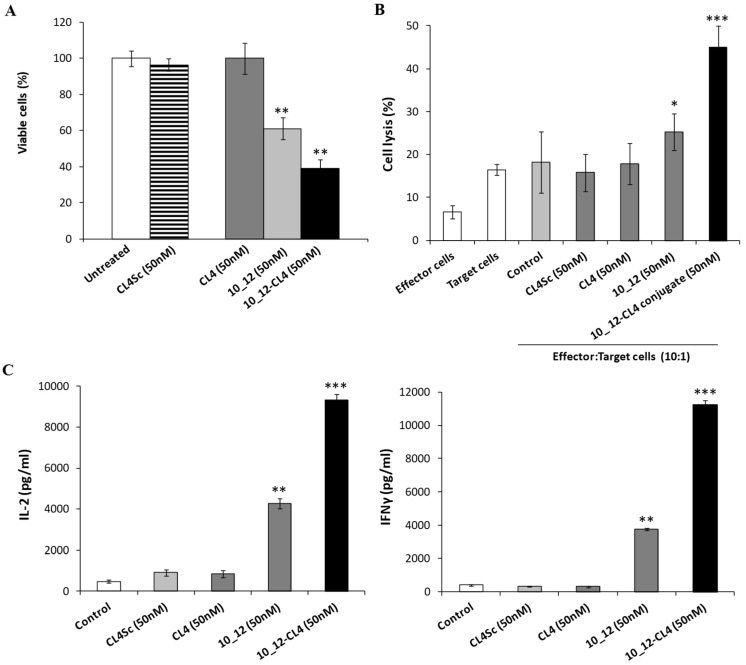
Cytotoxic effects of 10_12-CL4 conjugate on breast cancer cells co-cultured with lymphocytes. (**A**) SK-BR-3 cells were co-cultured with lymphocytes (effector:target ratio 10:1) and treated for 24 h with CL4 aptamer, 10_12 mAb, or 10_12-CL4 conjugate at the indicated concentrations. SK-BR-3 cell survival is expressed as percentage of viable treated cells with respect to untreated cells. (**B**) SK-BR-3 cell lysis, measured by the LDH release after the incubation with the indicated compounds, used at the concentration of 50 nM. The levels of LDH are expressed as percentage of lysis of treated cells with respect to the effects observed in co-cultures of tumor cells and lymphocytes in the absence of the drugs, used as controls. (**C**) IL-2 and IFNγ cytokine secretion levels (pg/mL) were measured by ELISA assays performed on cell supernatants. Untreated or treated cells with scrambled CL4 in the presence of lymphocytes were used as negative controls. Error bars depict means ± SD. *p-*values for the indicated mAbs relative to untreated cells are: *** *p* ≤ 0.001; ** *p* < 0.01; * *p* < 0.05.

As shown in Figure 9 and Supplementary Figure S6, the immunoconjugate 10_12-CL4 induced the death of tumor cells more efficiently than both the parental moieties, significantly increasing the secretion of IL-2 and IFNγ cytokines by lymphocytes, as well as LDH release by tumor cells.

## 3. Discussion

Oligonucleotide aptamers have gained attention as new promising drugs in the field of cancer therapy due to their binding ability, lack of immunogenicity, easy chemical synthesis, and adaptable modification procedures. However, there are no aptamers currently approved for cancer therapy, probably due to their rapid clearance, and only two aptamers (AS1411 against nucleolin and NOX-A12 against CXCL12) are in clinical trials for cancer therapy [43,64]. Nonetheless, aptamers are expected to have good penetration into tissues (e.g., solid tumors) due to their small size.

Larger size monoclonal antibodies are now among the most successful and important drugs for treating cancer patients due to their efficacy and safety [65], but despite their advantages in pharmacokinetics, they may exhibit limited penetration of solid tumors, compromising their therapeutic efficacy. Furthermore, despite their clinical and commercial success, it has become clear that more potent reagents are needed for cancer therapy and could be created from the antibody concept.

Bispecific antibodies could meet these needs, providing novel therapeutics with increased efficacy. Unlike conventional antibodies, bispecific mAbs are able to simultaneously bind to two different antigens and perform additional functions inconceivable for monoparatope antibodies. However bispecific antibodies suffer from certain issues that have limited their widespread use. First, the construction and production costs are too high for most researchers to easily develop therapeutic antibodies, due in part to the need of extensive DNA manipulation and recombinant protein purification steps [66]. Second, monoclonal antibodies are large (~150 kDa), which may hinder tumor tissue penetration [66,67], particularly when they should be conjugated in bispecific antibody-based constructs.

In order to optimize the specificity, tissue penetration, and half-life of a drug, which is critical for its efficacy, we have considered testing the conjugation of a small size aptamer (12 kDa) with an antibody targeting a different antigen to obtain novel bispecific constructs with lower molecular weight with respect to bispecifics consisting of two antibodies based reagents, thus increasing potential tumor penetration and target accessibility. On the other hand, the presence of the antibody in the construct increases the molecular size of the aptamer, thus overcoming its rapid clearance by renal filtration and enhancing its half-life in circulation [68,69].

To this aim we have constructed two different bispecific immunoconjugates, made up of the anti-EGFR aptamer (CL4) fused either with an anti-TAA (anti-ErbB2) compact antibody (Erb-hcAb) or with an immunomodulatory (anti-PD-L1) mAb (10_12), called Erb-hcAb-CL4 and 10_12-CL4, respectively. The mechanisms of action of Erb-hcAb, PD-L1_1 (the parental mAb of 10_12), 10_12, and CL4, previously clarified and reported in literature [44,45,46,47,58,60,70,71,72,73], can be combined to increase the antitumor effects, as they were found to be different but complementary. We demonstrated that both the immunoconjugates were easily and rapidly obtained, retained the biological functions of both the parental moieties, and acquired a more potent cytotoxic activity against tumor target cells by combining the biological properties of the two different targeting agents. Furthermore, the conjugation of the aptamer specific for EGFR with the immunomodulatory 10_12 mAb allowed also for the efficient redirection and activation of T cells against cancer cells, thus dramatically enhancing the cytotoxicity of the two conjugated partners.

We can conclude that the novel bispecific aptamer–antibody conjugates, reported here for the first time, are indeed endowed with the following advantages:

(1) Easy and quick procedures for production;
(2) Low cost of production;
(3) Simultaneous inhibition of two targets with enhanced antitumor effects;
(4) Increased specificity, due to the simultaneous targeting of two different antigens, for tumor cells that express both antigens, thus limiting unwanted side effects on normal cells expressing only one of the antigens; and
(5) Potential increased pharmacokinetic and pharmacodynamic properties due to the combination of the advantages of small size aptamer for increasing tumor penetration and target accessibility with those of the antibody, which allows for less rapid clearance by renal filtration and longer half-life in circulation.

A limitation of our work is based on the absence of in vivo studies, due to the low yields of purification of these immunoconjugates that should be improved with larger scale productions. However, previous findings in the literature support the novel concept that a monoclonal antibody can extend in vivo pharmacokinetics of an aptamer without reducing the tumor-targeting and anticancer effects of the aptamer [68], even though the reported immunoconjugate was not bispecific as it was made up of the cotinine-conjugated anti-VEGF pegaptanib aptamer and an anti-cotinine antibody.

Furthermore, a c-Met aptamer-Fc conjugate was generated as potential scaffold for cytotoxic payloads that resulted into specific targeting of lung cancer cells in in vitro settings. Differently from our conjugates, made up of two functional units, the above construct contained a fluorescent dye as a proof-of-principle cargo [69]. Nevertheless, this study remarks the keen interest in generating aptamer-based immunoconjugates to overcome the limited in vivo half-life and lack of effector functions of aptamers.

Finally, we think that our strategy has the potential to become a universal platform to extend this methodology to all the desired antibodies and aptamers by using linkage residues in the constant regions of the antibodies and easy conjugation protocols, and represents a proof of concept to open up the possibility to also develop other multispecific immunoconjugates that could combine one antibody with multiple aptamers with different specificities, thus allowing for simultaneous targeting of different TAAs without excessively increasing the size of the immunoconjugates.

## 4. Materials and Methods

### 4.1. Cell Cultures

MDA-MB-231 cells (from ATCC, Rockville, MD, USA) were cultured in Dulbecco’s Modified Eagle’s Medium (GibcoTM DMEM, Thermo Fisher Scientific, Waltham, MA, USA). SK-BR-3, LNCaP, MDA-MB-453, and NCI-N87 cells (from ATCC, Rockville, MD, USA) were cultured in RPMI 1640 (Gibco BRL, Life Technologies, Paisley, UK). MCF-7 cells were cultured in Modified Eagle’s Medium (MEM, Gibco, Life Technologies, Grand Island, NE, USA). Media were supplemented with 10% (vol/vol) heat-inactivated fetal bovine serum (FBS, Sigma-Aldrich, St. Louis, MO, USA), 50 UI/mL penicillin, 50 μg/mL streptomycin (50 μg/mL streptomycin), and 2 nM L-glutamine (all from GibcoTM, Thermo Fisher Scientific).

### 4.2. Isolation of Human Peripheral Blood Mononuclear Cells

Human PBMCs were isolated from blood of healthy donors by using Greiner Leucosep^®^ tube (Sigma-Aldrich) following the manufacturer’s instructions, and frozen in a solution containing 90% FBS and 10% dimethyl sulfoxide (DMSO) until use. Cryopreserved cell vials were gently thawed out by using RPMI 1640 medium (GibcoTM, Thermo Fisher Scientific) supplemented with 1% L-glutamine, 1% CTL-Wash™ (Cellular Technology Limited, Shaker Heights, OH, USA), and 100 U/mL Benzonase (Merck Millipore, Burlington, MA, USA). The collected hPBMCs were then washed by centrifugation, plated, and incubated overnight at 37 °C in R10 medium consisting of RPMI 1640 supplemented with 10% inactivated FBS, 1% L-glutamine, 50 U/mL penicillin, 50 μg/mL streptomycin, and 1% HEPES (GibcoTM, Thermo Fisher Scientific). After an overnight resting, the hPBMCs were collected in phosphate-buffered saline (PBS), counted by using the Muse^®^ Cell Analyzer (Merck Millipore), and resuspended at a density of 1 × 10^6^ cells/mL [60].

### 4.3. Aptamers

2′F-Py RNA aptamers (CL4, its corresponding versions containing a C6-NH_2_ or a biotin 5′ terminal modification, and the scrambled CL4Sc sequence) were purchased from TriLink Biotechnologies (Tebu-bio, San Diego, CA, USA) with purity above 95% (PAGE analysis, short wave). The sequences of the aptamers and handling protocols prior to each cell treatment were previously reported [46].

### 4.4. Antibody-Oligonucleotide Conjugation

The conjugation of Erb-hcAb or 10_12 mAb with CL4 aptamer was obtained by using an Antibody-Oligonuclotide Solulink Conjugation Kit (TriLink Biotechnologies).

Briefly, in the first step, the amino-terminated CL4 aptamer was labeled at 5′ with an aromatic aldehyde functional group (formylbenzamide, 4FB), by reaction with an amine-reactive NHS ester (S-4FB), following the manufacturer’s recommendations.

In the second step, Erb-hcAb or 10_12 antibody was modified with HyNic functional groups by incubating the antibody with S-HyNic reagent at RT for 2 h.

Then, 4FB-oligonucleotide was incubated with HyNic-modified antibody for 2 h at RT to allow for conjugation. After the incubation, the reaction mixture was buffer-exchanged by centrifugation at 1500× *g* for 2 min in a spin column (Solulink kit).

To obtain the conjugate, the reaction mixture was transferred into a tube containing magnetic beads and incubated for 40 min at RT, taking care to mix the beads every 10 min during the incubation period. The beads were then placed on a magnetic stand for discarding the supernatant. After several washes, the beads were resuspended into the Elution Buffer (Solulink kit) and incubated for 15 min, taking care to resuspend them every 5 min. After the last resuspension, the beads were placed on the magnet stand to elute the immunoconjugate. The final antibody-oligonucleotide conjugate concentration was determined by using a BCA protein assay Kit (Pierce, Perbio, Rockford, IL, USA).

### 4.5. Western Blotting Analysis of Cell Extracts

SK-BR-3, LNCaP, and MCF-7 cells grown in 100-mm dishes at 37 °C, were collected and centrifuged at 1200 rpm for 5 min; the cell pellets were lysed in a buffer containing 10 mM Tris-HCl (pH 7.4), 0.5% Nonidet-P-40, and 150 mM NaCl in the presence of protease inhibitors (Roche, IN, USA). After incubation on ice for 20 min, the extracts were clarified by centrifugation at 12,000 rpm for 15 min at 4 °C. Protein concentration was determined by the Bradford colorimetric assay (Sigma-Aldrich), and Western blotting analyses were performed by incubating the membranes with anti-ErbB2, anti-PD-L1, or anti-EGFR antibodies, followed by the HRP-conjugated secondary antibodies.  Figure S7 shows whole blots relative to the Western Blotting analyses shown in Figure 2.

### 4.6. ELISA Assays

ELISA assays were performed by using tumor cells or human activated lymphocytes. Briefly, tumor cells were plated into NuncTM flat-bottom 96-well plate at the density of 2 × 10^5^ cells/well, whereas human lymphocytes were plated into NuncTM flat-bottom 96-well plate at the density of 4 × 10^5^ cells/well after activation with anti-CD3/CD28 beads. After incubation with a blocking solution (PBS/BSA 6%) for 20 min at RT, cells were incubated in the absence or in the presence of Erb-hcAb compact antibody, 10_12 antibody, biotinylated CL4 aptamer, Erb-hcAb-CL4, or 10_12-CL4 immunoconjugates in PBS/BSA 3% buffer solution for 75 min at RT. After extensive washes with PBS 1× solution, the plates were incubated with HRP-conjugated Streptavidin (to detect the binding of CL4 aptamer) for 30 min at RT, or with anti-human IgG (H + L) HRP conjugate (to detect the binding of Erb-hcAB, 10_12 mAb, Erb-hcAb-CL4, and 10_12-CL4 agents) for 1 h at RT. Then, the plates were washed with PBS 1× and TMB reagent was added for 10 min before quenching with an equal volume of 1 N HCl. Absorbance at 450 nm was measured by the Envision plate reader (Perkin Elmer, 2102, San Diego, CA, USA).

For measuring the level of surface PD-L1 expression on tumor cells, SKBR3, LNCap, and MCF-7 cells (density of 2 × 10^5^) were incubated in triplicates in the absence or in the presence of the commercial anti-PD-L1 mAb (G & P Biosciences, Santa Clara, CA, USA) in PBS/BSA 3% buffer solution for 75 min at RT. After extensive washes with PBS 1× solution, the plates were incubated with anti-human IgG (H + L) HRP conjugate for 1 h at RT. Then the plates were treated as described above. Binding values were reported as the mean of at least three determinations obtained in three independent experiments.

### 4.7. Confocal Microscopy

SK-BR-3 and MDA-MB-453 cells (10^5^ cells/well in 24-well), previously seeded on a coverslip for 24 h, were incubated for 1 h with 500 nM of 10_12 or 10_12-CL4 conjugates in BlockAid™ Blocking Solution (Life Technologies) at RT. Then, cells were washed three times in PBS and fixed in PBS/PFA 4% for 20 min. For the fluorescence visualization of mAbs and conjugates, cells were incubated with FITC-labeled anti-human IgG antibody (Fluorescein (FITC) AffiniPure Goat Anti-Human IgG (H + L) 1:300, Jackson ImmunoResearch Laboratories Inc., Madison, WI, USA) for 1 h at RT and then washed three times with PBS. Finally, cells were incubated with 1.5 μM 4′,6-Diamidino-2-phenylindole (DAPI, D9542, Sigma-Aldrich) and mounted with glycerol/PBS. Samples were visualized by Zeiss LSM 700 META confocal microscopy equipped with a Plan-Apochromat 63×/1.4 Oil DIC objective.

### 4.8. Cell Viability Assays

To test the effects of the combinatorial treatments, SK-BR-3 and MDA-MB-453 cells were plated in 96-well flat-bottom plates at a density of 1.5 × 10^4^ cells/well, LNCaP and NCI-N87 cells were plated at the density of 7.5 × 10^3^ cells/well, and incubated for 16 h at 37 °C. CL4 (200–400 nM), Erb-hcAb. or 10_12 (50–100 nM) was added, alone or in combination, in the complete culture medium and incubated for 72 h. Viable cells were counted by the trypan blue exclusion test and cell survival was expressed as percent of viable cells in the presence of the drugs under test with respect to negative control cultures grown in the absence of the agents.

To test the effects of combinatorial treatments on co-cultures of tumor cells and lymphocytes, SK-BR-3 (1.5 × 10^4^ cells/well) and LNCaP (7.5 × 10^3^ cells/well) cells were plated in 96-well flat-bottom plates for 16 h. Then, lymphocytes, isolated from healthy donors, were added at effector:target ratio 10:1 in the absence or presence of CL4 aptamer (200 nM) or 10_12 mAb (50–100 nM), used alone or in combination, and incubated for 24 h at 37 °C. Controls included target cells incubated in the presence of effector cells, untreated or treated with the agents alone.

After the treatment, lymphocytes were removed and adherent cells were washed and counted by the trypan blue exclusion test. Cell survival was expressed as percent of viable cells in the presence of the treatments with respect to the cells left untreated or treated with CL4Sc aptamer, used as negative control.

To test the effects of Erb-hcAb-CL4 or 10_12-CL4 immunoconjugates on tumor cell viability, SK-BR-3 (1.5 × 10^4^cells/well), LNCaP (7.5 × 10^3^cells/well), and MCF-7 (1 × 10^4^ cells/well) cells were plated and incubated for 16 h. Then, Erb-hcAb-CL4 or 10_12-CL4 immunoconjugates and the parental Erb-hcAb, 10_12 mAb, or CL4 aptamer, used alone, were added at the concentration of 50–100 nM and incubated at 37 °C for 72 h. Viable cells were counted by the trypan blue exclusion test and cell survival was expressed as described above.

To test the effects of 10_12-CL4 immunoconjugates on co-cultures of tumor cells and lymphocytes, SK-BR-3 (1.5 × 10^4^ cells/well) cells were plated in 96-well flat-bottom plates for 16 h. Lymphocytes were added (effector:target ratio 10:1) in the absence or presence of 10_12-CL4 immunoconjugate, or the parental CL4 aptamer and 10_12 mAb at the concentration of 50 nM, and incubated for 24 h at 37 °C. After treatment, lymphocytes were removed and adherent cells were washed, counted by the trypan blue exclusion test, and cell survival was expressed as described above.

The images of the cells untreated or treated with the previously indicated compounds were acquired by a Leica Microsystems integrated microscope (DFC320, magnification 1:100).

### 4.9. Determination of Tumor Cells Lysis

Tumor cell lysis was determined by measuring the release of LDH in the supernatant of the co-cultures described above by LDH detection kit (Thermofisher Scientific), following the manufacturer’s recommendations. Lysis was calculated by measuring the fold increase of LDH in the presence of each mAb, with respect to the amount present in the supernatant of untreated cells, used as negative control.

Typically, cell survival and cytolysis values were obtained from at least three independent experiments in which triplicate counts were determined.

### 4.10. Effects of Immunomodulatory and Anti-TAA Drugs on Cytokine Release

To test the ability of the antibodies or the immunoconjugates to induce cytokine secretion, human lymphocytes were co-cultured with SK-BR-3 cells (Effector:Target ratio 10:1) into flat-bottom 96-well plate and incubated for 24 h in the absence or in the presence of 10_12-CL4 immunoconjugate (50 nM), or the parental 10_12 mAb (50 nM) or CL4 aptamer (200 nM), used alone or in combination. Untreated cells or cells treated with scrambled oligonucleotide sequence were used as negative controls. The levels of IL-2 or INFγ cytokines secreted in cell culture supernatant were measured by ELISA assays (DuoSet ELISA, R&D Systems, Minneapolis, MN, USA), according to the manufacturer’s recommendations. Concentration values were reported as the mean of at least three determinations.

### 4.11. Statistical Analyses

Error bars were calculated on the basis of the results obtained by at least three independent experiments. For these studies, differences between groups were assessed by Student’s *t*-test. Statistical significance was defined as *p* < 0.05.

## 5. Conclusions

We have generated novel bispecific aptamer–antibody conjugates that combine the favourable properties of the two different targeting antitumor agents, thus providing novel useful tools with improved therapeutic efficacy and specificity for cancer cell killing. We think that our strategy has the potential to become a universal platform to extend this methodology to all the desired antibodies and aptamers by using for linkage residues in the constant regions of the antibodies and easy conjugation protocols.

## Supplementary Materials

The following are available online at https://www.mdpi.com/2072-6694/11/9/1268/s1, Figure S1: Images of SK-BR-3 and LNCaP tumor cells treated with Erb-hcAb and CL4, Figure S2: Images of SK-BR-3 and LNCaP tumor cells treated with CL4 and anti-PD-L1 mAb, Figure S3: Cytotoxic effects of CL4 aptamer and 10_12 mAb on breast cancer cells co-cultured with lymphocytes, Figure S4: Effects of Erb-hcAb-CL4 conjugate on tumor cell survival, Figure S5: Effects of 10_12-CL4 conjugate on tumor cell survival, Figure S6: Cytotoxic effects of 10_12-CL4 conjugate on breast cancer cells co-cultured with lymphocytes, Figure S7: Whole Blots relative to the Western Blotting analyses shown in Figure 2.
