# Supplementary material for: Novel Human Bispecific Aptamer–Antibody Conjugates for Efficient Cancer Cell Killing
Source: Cancers (Basel). 2019 Aug 29;11(9):1268. doi: 10.3390/cancers11091268 (PMC6770524; doi:10.3390/cancers11091268)
Supplement: Supplementary file 1 [file cancers-11-01268-s001.pdf]

Supplementary Materials

Novel Human Bispecific Aptamer–Antibody Conjugates for Efficient Cancer Cell Killing

Margherita Passariello, Simona Camorani, Cinzia Vetrei, Laura Cerchia and Claudia De Lorenzo

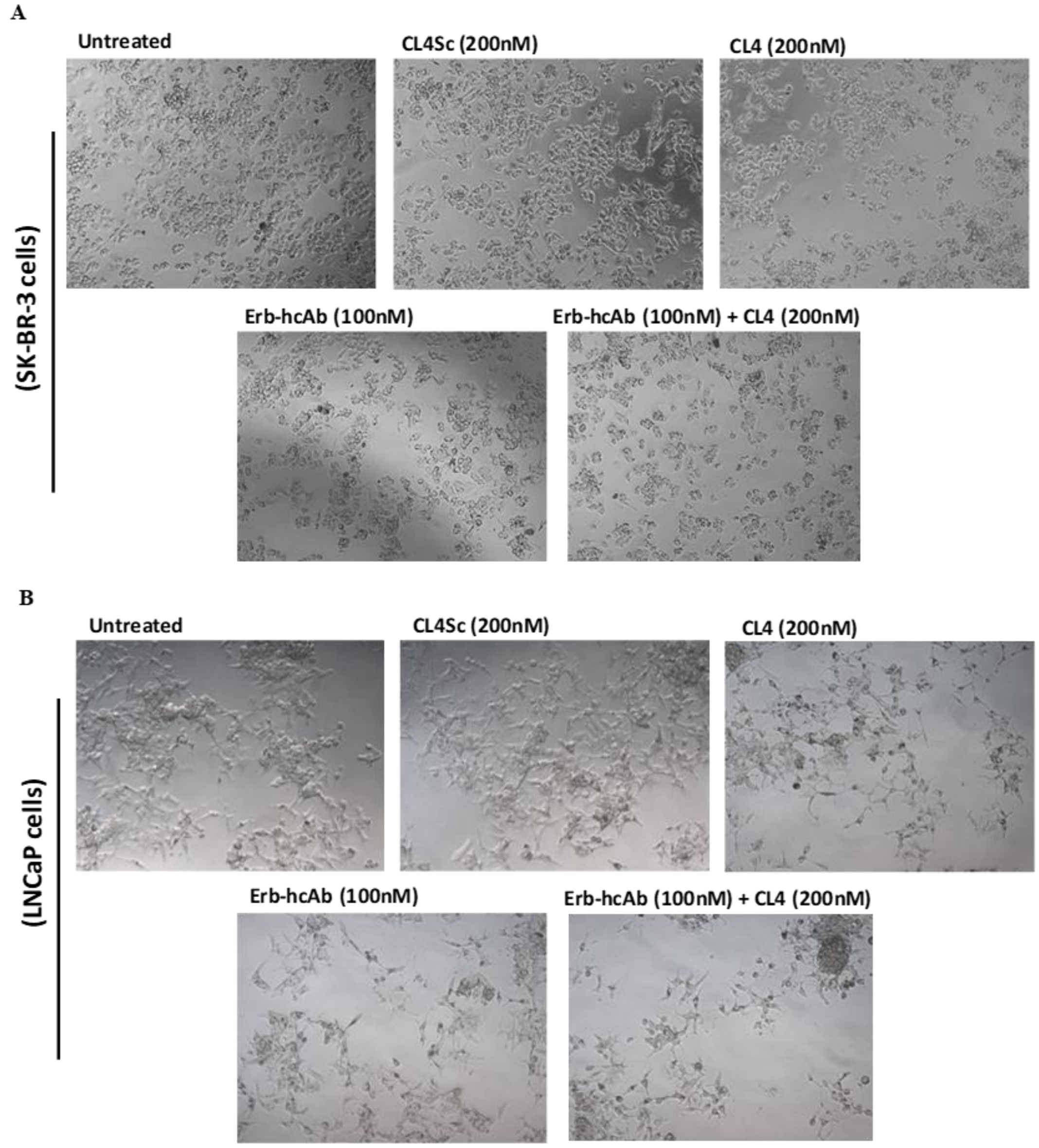

Figure S1. Images of SK-BR-3 and LNCaP tumor cells treated with Erb-hcAb and CL4. Representative images of SK-BR-3 (**A**) and LNCaP (**B**) cells treated for 72 h with Erb-hcAb or CL4, alone or in combination, at the indicated concentrations. The scrambled aptamer (CL4Sc) was used in parallel as a negative control.

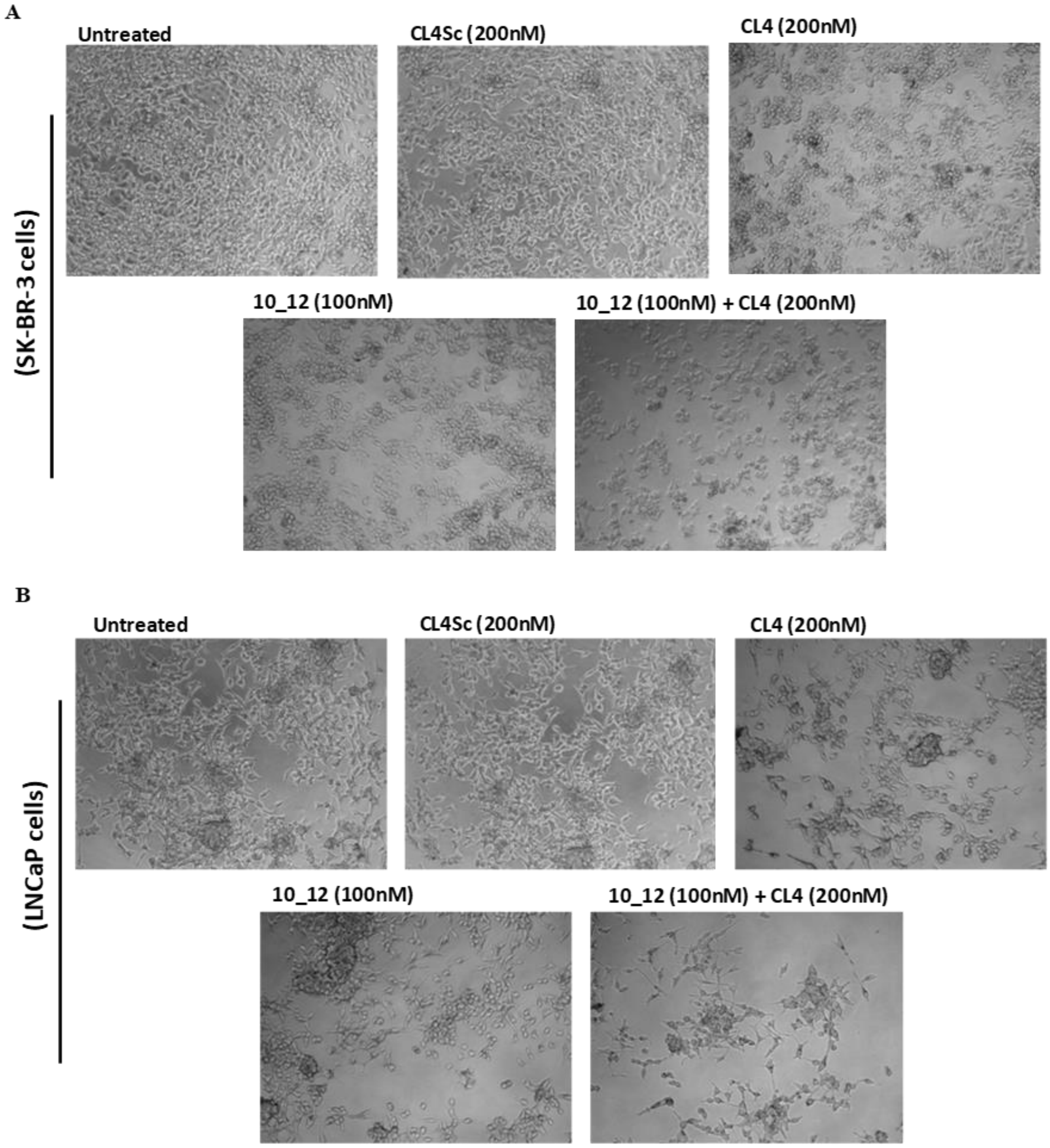

Figure S2. Images of SK-BR-3 and LNCaP tumor cells treated with CL4 and anti-PD-L1 mAb. Representative images of SK-BR-3 (**A**) and LNCaP (**B**) cells treated for 72 h with CL4 or 10_12 mAb, alone or in combination, at the indicated concentrations. CL4Sc was used in parallel as a negative control.

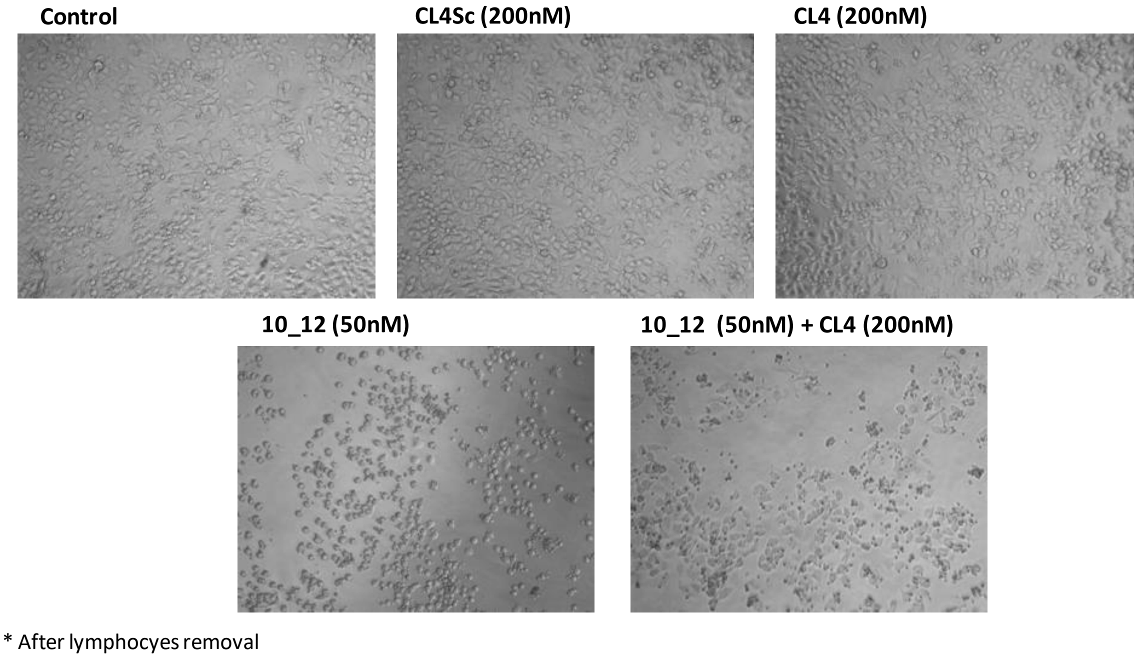

Figure S3. Cytotoxic effects of CL4 aptamer and 10_12 mAb on breast cancer cells co-cultured with lymphocytes. Representative images of SK-BR-3 cells co-cultured with lymphocytes (effector:target ratio 10:1) left untreated (Control) or treated for 24 h with CL4 or 10_12 used alone, or in combination, at the indicated concentrations. CL4Sc was used in parallel as negative control.

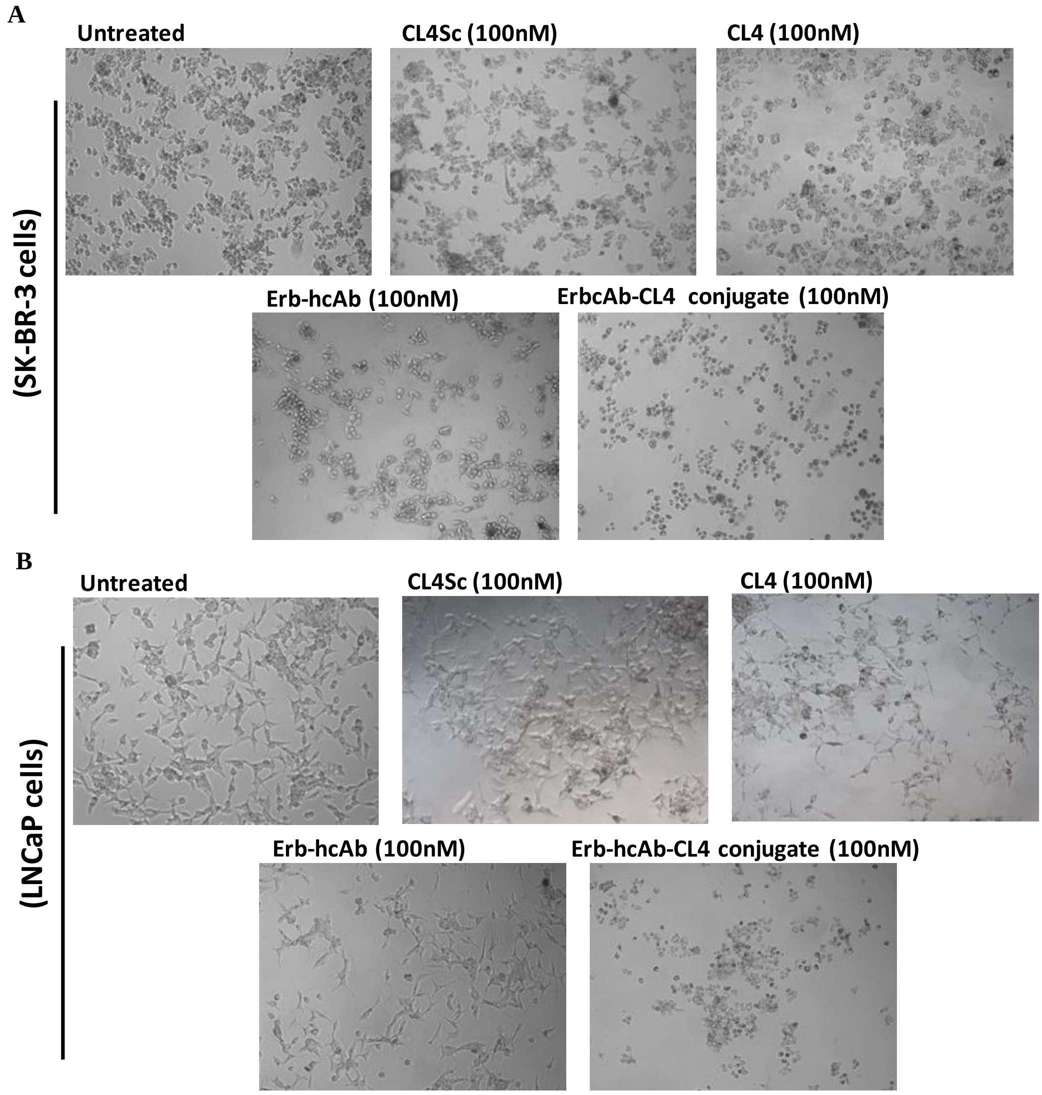

Figure S4. Effects of Erb-hcAb-CL4 conjugate on tumor cell survival. Representative images of SK-BR-3 (**A**) and LNCaP (**B**) cells untreated or treated with CL4, Erb-hcAb or or Erb-hcAb-CL4 conjugate at the indicated concentrations. CL4Sc was used in parallel as negative control.

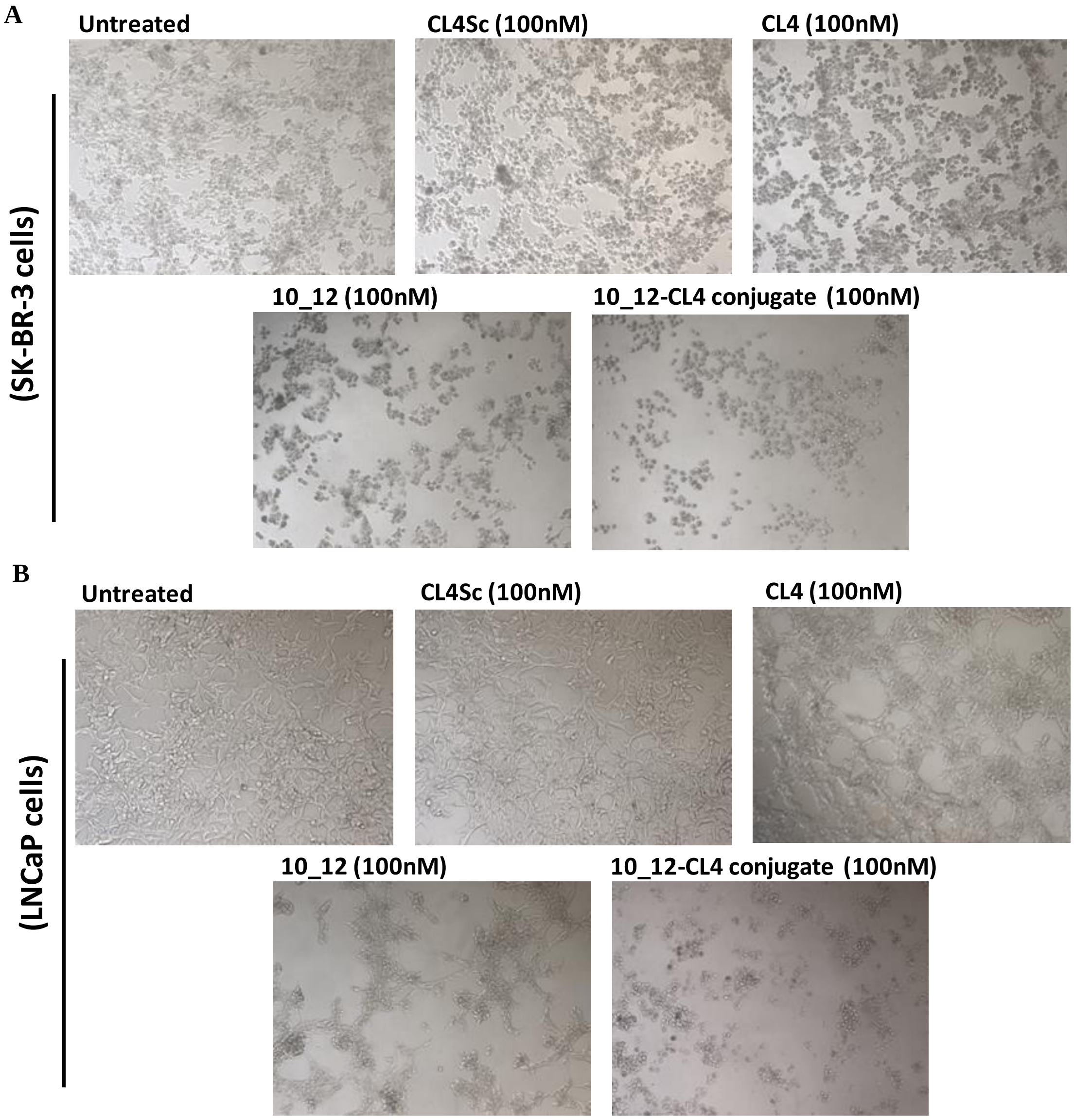

Figure S5. Effects of 10_12-CL4 conjugate on tumor cell survival. Representative images of SK-BR-3 (**A**) and LNCaP (**B**) cells untreated or treated with CL4, 10_12 or 10_12-CL4 conjugate at the indicated concnetrations. The scrambled aptamer was used in parallel as a negative control.

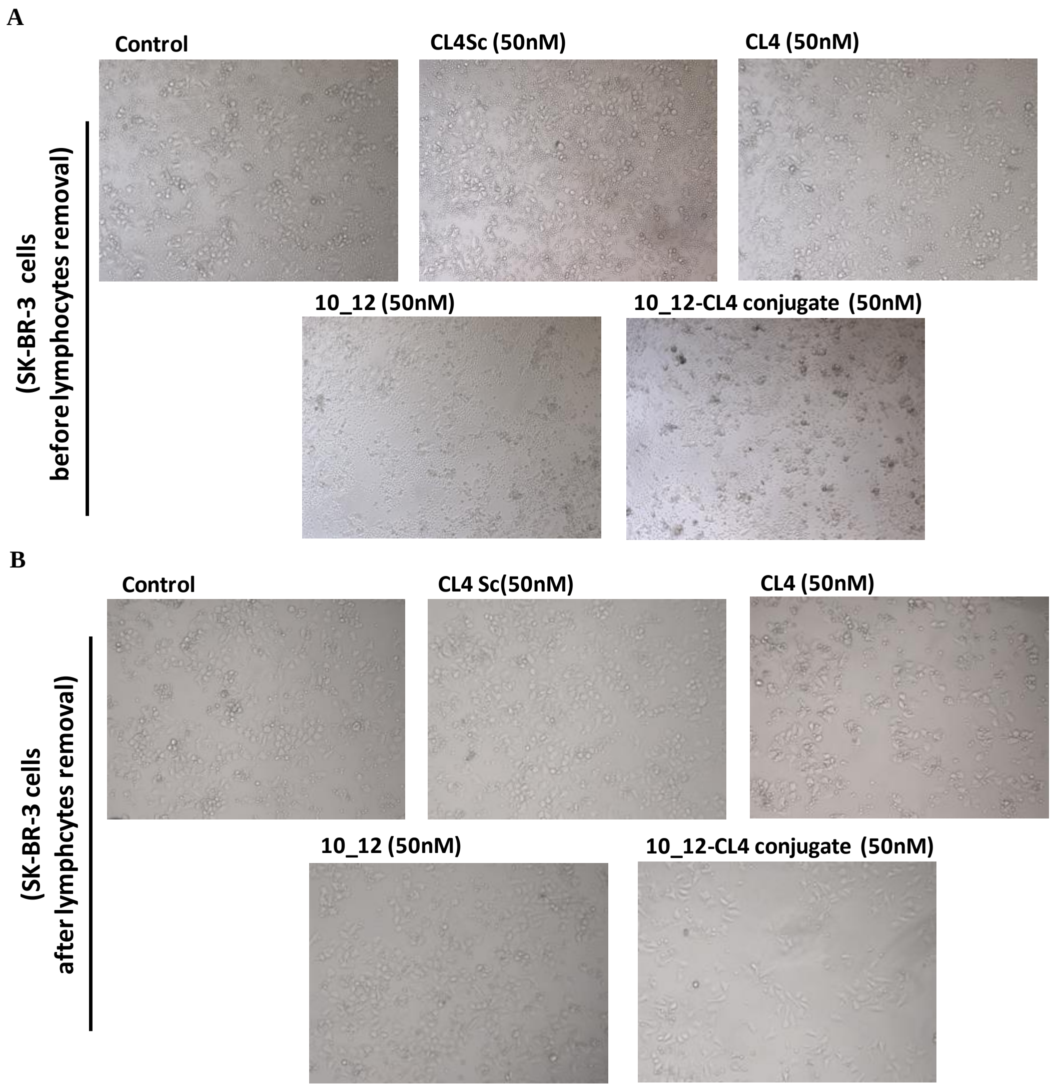

Figure S6. Cytotoxic effects of 10_12-CL4 conjugate on breast cancer cells co-cultured with lymphocytes. Representative images of SK-BR-3 cells co-cultured with lymphocytes (effector:target ratio 10:1), and treated for 24 hours with CL4 aptamer, 10_12 mAb, or 10_12-CL4 conjugate, at the indicated concentrations. The acquisition of images was carried out before (**A**) or after (**B**) lymphocytes removal. Cells untreated (Control) or treated with scrambled CL4 in the presence of lymphocytes were used as negative controls.

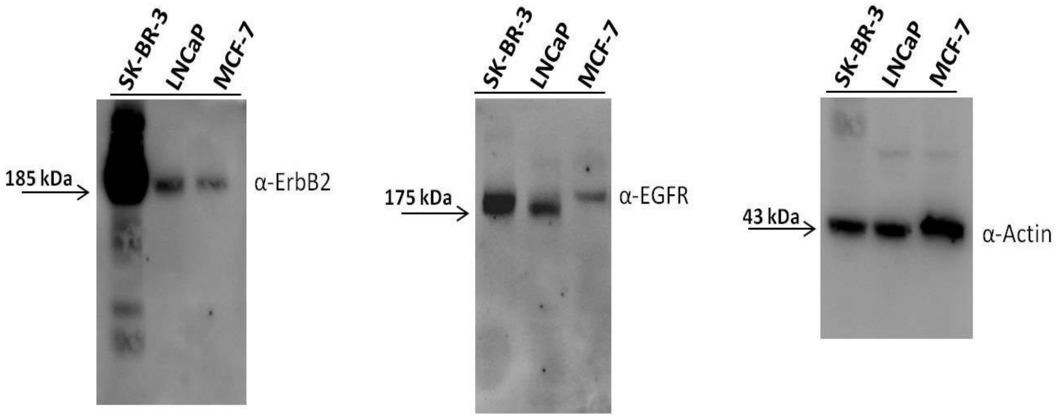

Figure S7. Whole Blots relative to the Western Blotting analyses shown in Figure 2.

© 2019 by the authors. Licensee MDPI, Basel, Switzerland. This article is an open access article distributed under the terms and conditions of the Creative Commons Attribution (CC BY) license (http://creativecommons.org/licenses/by/4.0/).
